# Delayed neuropsychological sequelae after carbon monoxide poisoning: predictive risk factors in the Emergency Department. A retrospective study

**DOI:** 10.1186/1757-7241-19-16

**Published:** 2011-03-17

**Authors:** Giuseppe Pepe, Matteo Castelli, Peiman Nazerian, Simone Vanni, Massimo Del Panta, Francesco Gambassi, Primo Botti, Andrea Missanelli, Stefano Grifoni

**Affiliations:** 1 Emergency Department, Careggi University General Hospital, Via delle Oblate 1, 50139, Florence, Italy; 2 Clinical Toxicology Unit, Careggi University General Hospital, Via delle Oblate 1, 50139, Florence, Italy

## Abstract

**Background:**

Delayed neuropsychological sequelae (DNS) commonly occur after recovery from acute carbon monoxide (CO) poisoning. The preventive role and the indications for hyperbaric oxygen therapy in the acute setting are still controversial. Early identification of patients at risk in the Emergency Department might permit an improvement in quality of care. We conducted a retrospective study to identify predictive risk factors for DNS development in the Emergency Department.

**Methods:**

We retrospectively considered all CO-poisoned patients admitted to the Emergency Department of Careggi University General Hospital (Florence, Italy) from 1992 to 2007. Patients were invited to participate in three follow-up visits at one, six and twelve months from hospital discharge. Clinical and biohumoral data were collected; univariate and multivariate analysis were performed to identify predictive risk factors for DNS.

**Results:**

Three hundred forty seven patients were admitted to the Emergency Department for acute CO poisoning from 1992 to 2007; 141/347 patients participated in the follow-up visit at one month from hospital discharge. Thirty four/141 patients were diagnosed with DNS (24.1%). Five/34 patients previously diagnosed as having DNS presented to the follow-up visit at six months, reporting a complete recovery. The following variables (collected before or upon Emergency Department admission) were associated to DNS development at one month from hospital discharge in the univariate analysis: CO exposure duration >6 hours, a Glasgow Coma Scale (GCS) score <9, seizures, systolic blood pressure <90 mmHg, elevated creatine phosphokinase concentration and leukocytosis. There was no significant correlation with age, sex, voluntary exposure, headache, transient loss of consciousness, GCS between 14 and 9, arterial lactate and carboxyhemoglobin concentration. The multivariate analysis confirmed as independent prognostic factors GCS <9 (OR 7.15; CI 95%: 1.04-48.8) and leukocytosis (OR 3.31; CI 95%: 1.02-10.71).

**Conclusions:**

Our study identified several potential predictive risk factors for DNS. Treatment algorithms based on an appropriate risk-stratification of patients in the Emergency Department might reduce DNS incidence; however, more studies are needed. Adequate follow-up after hospital discharge, aimed at correct recognition of DNS, is also important.

## Background

Carbon monoxide (CO) is a common cause of poisoning, resulting in more than 40.000 cases per year in the United States [1,2]. In Italy, estimated incidence is about 6.000 cases per year (resulting in more than 350 deaths/year) [3]; in the United Kingdom, about 50 people annually die because of CO-poisoning [4]. The real incidence is underestimated because the majority of cases go unrecognized or unreported. Presenting symptoms are notoriously aspecific (headache, asthenia, nausea, vomiting, transient loss of consciousness, altered mental status, coma) and correct diagnosis is a challenge for the emergency physician. Mortality among patients admitted to the hospital is low, and prevention of delayed neuropsychological sequelae (DNS) has become the main goal of treatment. DNS usually develop within some weeks after an initial complete clinical recovery from acute poisoning. The reported incidence varies widely from 3 to 40%, because of the lack of established diagnostic criteria [1-16]. Most frequently described sequelae include a broad spectrum of neurological deficits, cognitive impairments and affective disorders [Table 1] [1-16]. DNS gradually resolve over the the first months but can be permanent in about 25% of cases [1-16]; patients with persistent complaints are usually referred to a specialist for long-term therapy. Patient's initial presentation does not predict the development of DNS with certainty, but some variables have been associated to DNS: older age, duration of exposure to CO, longer time to treatment, transient loss of consciousness, coma, abnormal results on early neuropsychometric testing, severe metabolic acidosis, high arterial lactate level, increased serum levels of neuron-specific enolase (NSE), increased levels of myelin basic protein in the cerebrospinal fluid and early head computed tomography (CT) or magnetic resonance (MRI) abnormalities [1,2,5-18]. The preventive role of hyperbaric oxygen (HBO), administered in the acute setting, has been evaluated in several trials [6-13] and one metanalysis [14] but results are controversial. In particular there are uncertainties about early identification of patients for whom HBO is most likely to benefit [1,2,15,16]. Treatment algorithms are variable but it is currently recommended to consider HBO administration to patients presenting at least one of these signs or symptoms before or upon hospital admission: coma, seizures, transient loss of consciousness, altered mental status, abnormal neurological signs, myocardial injury and severe metabolic acidosis. It is also considered a therapeutic option for pregnant women and young children [1,2,15,16]. An elevated carboxyhemoglobin level (>25%; >15% during pregnancy) is still considered an appropriate indication in some treatment algorithms [2]; however, this value does not reflect poisoning severity and even high values have not been associated to DNS [1,15,16]. A correct identification of the patients at risk in the Emergency Department (ED) might be useful in order to improve treatment, prevent DNS and allow an appropriate medical follow-up after hospital discharge. We conducted a retrospective study to identify which early clinical and instrumental data, available to the emergency physician, predict the development of DNS.

**Table 1:** Signs and symptoms of delayed neuropsychological sequelae

Neurological sequelae	Cognitive and psychological sequelae
Parkinson-like syndromes	Concentration deficit
Gait and motor disturbances	Memory loss
Bradykinesia	Cognitive impairment
Intention tremor	Dementia
Myoclonus	Personality changes
Dyspraxia	Anxiety
Dysphasia	Extreme emotional lability
Ataxia	Psychosis
Postural instability	Depression
Vertigo	Mania
Cortical blindness	Insomnia
Hearing loss, tinnitus	
Chorea	
EEG abnormalities	
Epilepsy	
Peripheral neuropathies	
Recurrent headache	
Fecal/urinay incontinence	

References: [1-18]

## Methods

A retrospective medical record review was conducted on all CO-poisoned patients admitted to the ED of Careggi University General Hospital from 1992 to 2007. Carboxyhemoglobin levels were measured by an arterial blood gas analyzer with a CO-oximeter. A carboxyhemoglobin level at admission greater than 5% in non-smokers and >10% in smokers was considered diagnostic [1,2]; patients with a lower level but with suggestive symptoms and a confirmed exposure to CO (through the use of ambient CO detectors by emergency medical services or the presence of relatives or cohabitants with diagnosed poisoning) were also included. Patients with mixed poisoning (carbon monoxide and drugs, alcohol or cyanide) were excluded. Every patient received high-flow supplemental oxygen in the acute setting. In our hospital CO-poisoned patients are currently treated with HBO according to specific criteria [3] (pregnant women, children <2 years old, presence of GCS <9, seizures, transient loss of consciousness, altered mental status, abnormal neurological signs, evidence of myocardial injury and severe metabolic acidosis - pH <7.2), but an hyperbaric chamber became available only in 2001. Consequently, every patient admitted before 2001, despite poisoning severity, was treated with normobaric oxygen (NBO), defined as supplemental oxygen administration in the highest concentrations available. The presence of the following variables collected before or upon ED admission was considered: age, sex, duration of CO exposure (>or <6 hours), voluntary or accidental exposure, coma (defined as Glasgow Coma Scale (GCS) <9), transient loss of consciousness, seizures, altered level of consciousness (defined as GCS between 9 and 14), headache, postural instability (defined as positive Romberg's sign), systolic blood pressure <90 mmHg, evidence of myocardial injury (defined as electrocardiographic signs of ischaemia/arrythmias and/or cardiac enzymes elevation), dyspnea, carboxyhemoglobin level, pH, paCO2, paO2 and lactate levels in an arterial blood sample at admission. Haemoglobin (Hb), platelet count (Plt), white blood cell count (WBC), troponin I and creatine phosphokinase (CPK) concentration in a venous blood sample obtained within 6 hours from admission were also collected; treatment modality (HBO or NBO) was also considered. Every patient was invited to participate in three follow-up visits in a dedicated outpatient clinic at one, six and twelve months from hospital discharge. During the visit a complete neurological exam was performed and Folstein Mini Mental Status Examination (MMSE) was administered. DNS were defined as the presence of neurological, cognitive or affective disorders [Table 1], developed after hospital discharge. Neuroradiological imaging (head CT/head MRI or SPECT) was proposed to patients suffering from DNS.

### Statistical analysis

We investigated the prognostic value of the analyzed variables for the occurrence of DNS development at one month from hospital discharge. We performed univariate analysis in which the variables were compared between patients with DNS and patients with a complete clinical recovery. We used Student's t-test and analysis of variance for continuous variables and Fisher's test for categorical variables. HBO therapy was considered a categorical variable (yes/not). Univariate analysis was performed using a logistic regression model to estimate the odds ratio (OR) of developing DNS, along with 95% confidence interval (CI). Variables significantly related to DNS development were selected, multivariate analysis was performed (stepwise forward regression model) and OR estimated. Then a comparison was performed (using Student's t-test and analysis of variance for continuous variables and Fisher's test for categorical variables) between the group of patients treated with HBO and the group treated with NBO. The potential benefit of HBO (in terms of DNS incidence reduction at one month) was investigated. Only patients presenting signs or symptoms currently considered indications for HBO in our hospital were included; patients treated with NBO because of the absence at admission of signs and symptoms considered indications for HBO were excluded. For all tests, two-sided p-values less than .05 were considered statistically significant. In text and tables, variables are expressed as mean ± standard deviation (SD). Statistical analysis have been conducted using the SPSS statistical package software, v. 14.0, SPSS, Chicago, Illinois).

## Results

Three hundred fifty seven CO-poisoned patients presented to the ED from 1992 to 2007 (mean age: 41 ± 21 years; males: 160). Ten patients were excluded because of mixed poisoning with drugs (mainly benzodiazepines and antidepressants) or alcohol. More than 65% of patients were transported to the ED by emergency medical services. Presenting clinical characteristics were aspecific; the signs and symptoms most commonly reported are listed in Table 2. Evidence of myocardial injury was present in 17.2% of patients; systolic blood pressure <90 mmHg in 1.7%. Severe metabolic acidosis (arterial pH <7.2), dyspnea or cardiac chest pain (defined as the presence of chest pain associated to electrocardiographic signs of ischaemia/arrythmias and/or cardiac enzymes elevation) were present in less than 6.5% of patients. The cherry pink colour of the skin, traditionally considered distinctive of CO poisoning, was present in less than 1% of patients. Sixteen patients (4.6%) were asymptomatic but with diagnostic carboxyhemoglobin levels. Mean carboxyhemoglobin level at admission was 24.8 ± 10%; this value did not correlate with poisoning clinical severity. Two elderly patients died in the ED (0.57%), both presenting coma, myocardial injury and haemodynamic instability. Mean duration of hospitalization was 3.7 ± 2.5 days. Ninety-six/347 (27.6%) patients were treated with HBO, in 3 cases because of pregnancy and in 3 cases because of age <2 years. The remainder patients (251/347, 72.4%) were treated with NBO; patients admitted before 2001 received NBO despite the presence of signs and symptoms currently considered indications for HBO. One hundred forty one/347 (40.6%) patients were evaluated at 30 days from discharge (mean age: 42 ± 21 years; males: 64); DNS were diagnosed in 34/141 (24.1%). The remainder patients completely recovered and resumed their former occupation after hospital discharge. Only 13/141 patients (9.2%) participated to the follow-up visit at six months from hospital discharge. Five were previously diagnosed as having DNS and reported a complete recovery. None of the patients presented to the visit at one year. Most frequent sequelae consisted of mild cognitive dysfunctions: memory and concentration impairment (25/34), difficulty in calculating (9), writing and reading (5). Mean MMSE score was 24.1 ± 1.7. Fifteen patients developed recurrent headache. Five patients complained of affective disorders (depression or anxiety) diagnosed by a specialist after hospital discharge; three patients suffered from insomnia. Abnormal neurological signs were rare (7/34), mainly related to cerebellar and basal ganglia injury: postural instability, rigidity, bradykinesia, gait disturbances, intention tremor, impaired coordination and dysarthria. Dyspraxia, dysphasia and anosmia developed respectively in three, two and one patient. A Parkinson-like syndrome with severe cognitive impairment and urinary incontinence developed in another case (a 66 years-old male presenting coma and haemodynamic instability at admission). The univariate analysis identified the variables associated to DNS development: CO exposure duration >6 hours (p = 0.03), GCS <9 at admission (p = 0.002), seizures (p = 0.001) and systolic blood pressure <90 mmHg (p = 0.04). A CPK concentration >160 U/L (normal range: 20-160 U/L) and leukocytosis (WBC >10.000/microliter) within 6 hours from admission also resulted associated (p = 0.01 and p = 0.004). There was no correlation with age, sex, suicide attempt, duration of exposure, transient loss of consciousness, GCS between 14 and 9, metabolic acidosis and blood gas analysis abnormalities, in particular carboxyhemoglobin level at admission. HBO administration (55/141 patients, 39%) did not result associated to a significant reduction in the DNS incidence at one month (p = 0.36) [Table 3]. The multivariate analysis confirmed as independent variables predictive of risk GCS <9 (OR 7.15; CI 95%: 1.04-48.8) and leukocytosis within 6 hours from admission (OR 3.31; CI 95%: 1.02-10.71) [Table 4]. The inclusion of categorical variable "HBO therapy" in the previous multivariate analysis confirmed the lack of an association between DNS and HBO, confirming at the same time the correlation with leukocytosis and GCS <9. Among the 141 patients who presented to the first follow-up visit, 108 presented at admission signs or symptoms currently considered indications for HBO in our hospital. Patients admitted before 2001 were treated with NBO, because of the unavailability of an hyperbaric chamber in our hospital (53/108, 49%). The remainder patients (55/108, 50.9%) were treated with HBO. The base-line characteristics of the two groups are reported in Table 5. The DNS incidence at one month was 26.8% (29/108 patients), the difference between the two groups was not statistically significant (23.6% in the HBO group vs. 30.1% in the NBO group; p = 0.44). The univariate analysis did not show a significant association between HBO administration and DNS incidence reduction (OR 0.72; IC 95%: 0.30-1.68). Among patients who suffered from DNS, 5(14.7%) did not present at admission signs or symptoms currently considered indications for HBO.

**Table 2:** Presenting signs and symptoms of acute carbon monoxide poisoning

Signs/symptoms in the Emergency Department	n = 347	%
Headache	203	58.5%

Transient loss of consciousness	142	40.9%

Sinus tachycardia	90	25.9%

Nausea/vomiting	87	25.0%

Asthenia	70	20.1%

Dizziness	70	20.1%

Palpitations	64	18.4%

Evidence of myocardial injury	60	17.2%

GCS between 14 and 9	53	15.2%

Postural instability	37	10.6%

GCS <9	29	8.3%

Seizures	24	6.9%

Dyspnea	22	6.3%

Severe metabolic acidosis (pH <7.2)	15	4.3%

Cardiac chest pain	6	1.7%

Systolic blood pressure <90 mmHg	6	1.7%

347 patients

**Table 3:** Base-line characteristics of patients and association between variables and DNS development

Variables (before or upon ED admission)	Patient number = 141 mean ± SD	Non-DNS group at one month from hospital discharge (patient n = 107) mean ± SD	DNS group at one month from hospital discharge (patient n = 34) mean ± SD	p value
Age (years)	42 ± 21	41.7 ± 21.7	40.4 ± 15.5	>.05

Sex (male)	64 (45.3%)	53 (49.5%)	11 (32.3%)	>.05

Duration of exposure >6 hours	54 (38.2%)	36 (33.6%)	18 (52.9%)	.03

Suicide attempt	5 (3.5%)	3 (2.8%)	2 (5.8%)	>.05

Hb (g/dL)	14 ± 1.6	14.1 ± 1.16	13.7 ± 1.9	>.05

WBC (per microlitre)	9.87 ± 3.7	9.100 ± 2.900	11.900 ± 4.900	.004

Plt (per microlitre)	234.300 ± 58.300	236.000 ± 61.700	228.000 ± 47.000	>.05

Troponin I (ng/mL) (n = 43)	0.72 ± 3.25	0.88 ± 3.88 (n = 30)	0.33 ± 0.66 (n = 13)	>.05

CPK (U/L) (n = 127)	255 ± 763	159 ± 221 (n = 97)	572 ± 1506 (n = 30)	.01

Lactate (mmol/L) (n = 82)	1.77 ± 1.46	1.77 ± 1.41 (n = 62)	1.76 ± 1.34 (n = 20)	>.05

pH	7.41 ± 0.07	7.40 ± 0.07	7.41 ± 0.07	>.05

paCO2 (KPa)	4.78 ± 0.87	4.82 ± 0.89	4.62 ± 0.74	>.05

paO2 (KPa)	15.33 ± 1.09	14.93 ± 7.34	16.53 ± 10.45	>.05

GCS <9	15 (10.6%)	6 (5.6%)	9 (26.4%)	.002

Transient loss of consciousness	75 (53.1%)	53 (49.5%)	22 (64.7%)	>.05

Seizures	14 (9.9%)	5 (4.6%)	9 (26.4%)	.001

Systolic blood pressure <90 mmHg	4 (2.8%)	1 (0.9%)	3 (8.8%)	.04

Postural instability	37 (26.2%)	26 (24.2%)	11 (32.3%)	>.05

Headache	79 (56%)	60 (56%)	19 (55.8%)	>.05

GCS between 14 and 9	29 (20.5%)	26 (24.2%)	3 (8.8%)	>.05

Severe metabolic acidosis (pH <7.2)	11 (7.8%)	6 (5.6%)	5 (14.7%)	>.05

Dyspnea	6 (4.2%)	4 (3.7%)	2 (5.8%)	>.05

Mean carboxyhemoglobin level at admission (%)	26.3 ± 10	26 ± 10.2	26.4 ± 10.5	>.05

Evidence of myocardial injury	33 (23.4%)	23 (21,4%)	10 (29,4%)	>.05

HBO therapy	55 (39%)	44 (41.1%)	11 (32.3%)	>.05

141/347 (40,6%) patients participated in the follow-up visit at one month from hospital discharge.

**Table 4:** Predictive risk factors for DNS development

Variables	Univariate analysis Odds ratio (confidence interval: 95%)	Multivariate analysis Odds ratio (confidence interval: 95%)
Systolic blood pressure <90 mmHg	10.25 (1.40-73.68)	1.34 (0.04-40.85)

Seizures	7.34 (2.53-22.28)	1.65 (0.18-14.85)

GCS <9	6.06 (2.04-17,95)	7.15 (1.04-48.8)

Leukocytosis (WBC >10.000/microlitre)	3.13 (1.31-7.95)	3.31 (1.02-10.71)

Duration of exposure >6 hours	2.13 (0.87-5.24)	1.28 (0.09-2.9)

CPK >160 U/L	1.14 (0.49-2.60)	1.02 (0.4-1.64)

Odds ratios for the association of variables with DNS development at one month from hospital discharge. Univariate and multivariate analysis.

**Table 5:** Comparison between DNS incidence among patients treated with NBO and patients treated with HBO. Base-line characteristics of patients (n = 108)

Variables (before or upon ED admission)	Patient number = 108 mean ± SD	OTI group (patient n = 55) mean ± SD	OTN group (patient n = 53) mean ± SD	p value
Age (years)	44 ± 20	40.6 ± 20.4	48.2 ± 21.5	>.05

Sex (male)	50 (50.9%)	23 (41.8%)	27 (50.9%)	>.05

Duration of exposure > 6 hours	41 (37.9%)	27 (49.9%)	14 (26.4%)	.01

Suicide attempt	4 (3.7%)	3 (5.4%)	1 (1.8%)	>.05

Hb (g/dL)	14 ± 1.7	13.8 ± 1.6	14 ± 1.8	>.05

WBC (per microlitre)	10.100 ± 3.890	10.300 ± 4.100	9.800 ± 3.500	>.05

Plt (per microlitre)	226.000 ± 50.000	220.100 ± 45.000	233.000 ± 54.300	>.05

Troponin I (ng/mL) (n = 36)	0.85 ± 3.54	0.98 ± 3.88 (n = 30)	0.22 ± 0.44 (n = 6)	>.05

CPK (U/L) (n = 93)	298 ± 867	385 ± 1149 (n = 50)	195 ± 265 (n = 43)	>.05

Lactate (mmol/L) (n = 65)	1.94 ± 1.6	2.05 ± 1.78 (n = 43)	1.75 ± 1.1 (n = 22)	>.05

pH	7.40 ± 0.08	7.41 ± 0.06	7.39 ± 0.1	>.05

paCO2 (KPa)	4.81 ± 0.94	4.73 ± 0.6	4.89 ± 1.21	>.05

paO2 (KPa)	15.8 ± 8.78	16.7 ± 8.0	14.7 ± 9.4	>.05

GCS <9	15 (13.8%)	9 (16.3%)	6 (11.3%)	>.05

Transient loss of consciousness	75 (69.4%)	42 (76.3%)	33 (62.2%)	>.05

Seizures	14 (12.9%)	12 (21.8%)	2 (3.7%)	.008

Systolic blood pressure <90 mmHg	4 (3.7%)	1 (1.8%)	3 (5.6%)	>.05

Postural instability	32 (29.6%)	14 (25.4%)	18 (33.9%)	>.05

Headache	54 (50%)	31 (63.6%)	23 (43.3%)	>.05

GCS between 14 and 9	29 (26.8%)	14 (25.4%)	15 (28.3%)	>.05

Severe metabolic acidosis (pH <7.2)	11 (10.1%)	5 (9%)	6 (11.3%)	>.05

Dyspnea	5 (4.6%)	2 (3.6%)	3 (5.6%)	>.05

Mean carboxyhemoglobin level at admission (%)	27.7 ± 9.6	25.9 ± 9.41	29.5 ± 9.7	>.05

Evidence of myocardial injury	33 (30.5%)	14 (25.4%)	19 (35.8%)	>.05

DNS incidence at one month	29 (26.8%)	13 (23.6%)	16 (30.1%)	>.05

Only patients presenting at admission signs and symptoms currently considered indications for HBO therapy in our hospital were selected.

## Discussion

In our study, DNS resulted a relatively frequent complication of CO poisoning (24.1% incidence at 30 days from hospital discharge) although clinically mild in most cases. Mortality among CO poisoned patients admitted to the hospital was low (0.57%). We identified several clinical and instrumental data associated to DNS development. Prolonged CO exposure, seizures and GCS <9 are expression of severe poisoning with high probability of neurological injury. A systolic blood pressure <90 mmHg, although significantly correlated with DNS development, was present in only four patients, a number too low to draw definitive conclusions. Increased CPK concentration (>160 U/L) indicates both CO-mediated muscle necrosis and rhabdomyolysis in comatose patients lying on the floor for a long time. Leukocytosis is a condition that has never been associated to DNS, but this result can be interpreted considering the role of activated leukocytes in the pathogenesis of neurological injury, mainly attributable to oxidative stress and inflammation, than to hypoxia [19,20]. However, considering the limited difference in white blood cell count between the two groups of patients (non-DNS and DNS), our finding does not seem to have a clinically relevant impact. Furthermore, whether antiinflammatory therapy could prevent DNS is unknown [1,16]. On the other hand, we did not find an association between DNS and some variables previously correlated with neurological damage in other studies [5-18]; some of them are current indications for HBO (for example, carboxyhemoglobin level, transient loss of consciousness and GCS between 9 and 14). Carboxyhemoglobin level at admission has a fundamental diagnostic role, but the lack of prognostic value was confirmed in our study. Carboxyhemoglobin level is influenced by several factors, such as pre-hospital oxygen administration and time elapsed between exposure cessation and hospital admission. At the moment, the preventive role of HBO has not been clearly demonstrated, and whether to use HBO and in which patients remains controversial. It would be useful to revise and implement the current indications for HBO, considering the presence or the lack, in the acute setting, of reliable predictors of DNS. Furthermore, it would be interesting to evaluate the potential benefit of HBO for patients with signs and symptoms, associated to DNS development, not currently considered indications for this therapy. The lack of a statistically significant association between HBO administration and DNS incidence reduction was also evident in our study, although a trend in this direction was present [Table 4,5]. The fact that 5/34 patients who suffered from DNS did not present, at admission, signs or symptoms currently considered indications for HBO could be an interesting result. However, these results were influenced by several limitations: patients treated with HBO presented, on average, a more severe poisoning, even excluding the 33/141 patients suffering from mild poisoning who were treated only with NBO because of the absence at admission of signs and symptoms considered indications for HBO [Table 5]; hyperbaric protocols adopted by our hyperbaric medicine specialists were not equal for all patients (mainly because of the lack of specific universally accepted guidelines) and even NBO therapy was variable (in terms of duration and oxygen concentration). Additionally, it was not possible to acquire information about prehospital treatment (in terms of supplemental oxygen and/or drugs administration).

Our study presents some other limitations. First of all, patients' low compliance with follow up (in particular at six and twelve months) influenced the reported incidence and clinical characteristics of DNS. The reported incidence was probably overestimated, considering that compliance to follow-up (especially to the visit at one month) was probably higher in patients suffering from DNS. The even lower compliance to the next follow-up visits can be explained considering both the group of patients who completely recovered and the group of symptomatic patients who referred to a specialist; both groups interrupted the follow-up program. Additionally, this limitation did not permit us to identify late-onset DNS and to evaluate their duration. Our diagnostic accuracy was influenced by several factors. First of all, we adopted diagnostic criteria different from other studies because of the lack of established ones. Furthermore, only a few patients with DNS were evaluated with CT, MRI or single-photon emission computed tomography during follow-up. Several abnormal radiological findings associated to DNS have been reported in literature, in particular injuries of the cerebral cortex, white matter, hippocampus and globus pallidus [1,2,16,17], but in our study, the majority of patients consulted a specialist and underwent neuroradiological imaging privately, interrupting the follow-up.

## Conclusions

Acute CO poisoning is still a challenge in emergency medicine: correct diagnosis, optimal treatment and DNS prevention are fundamental goals. There are no established criteria, if any, for determining the risk for DNS. Appropriate information about DNS should be given to every patient at the moment of hospital discharge, to allow prompt recognition of sequelae. In our study, sequelae were common after recovery from acute CO poisoning. We identified several potential predictive risk factors for DNS development. Early risk-stratification of patients in the Emergency Department might permit an improvement in quality of care, in terms of DNS prevention. No clear evidences are available whether or not HBO therapy is beneficial in preventing DNS, and the indications for this treatment in the acute setting remain controversial. Further research is needed to better define the optimal management of CO poisoning.

## Competing interests

The authors declare that they have no competing interests.

## Authors' contributions

GP, MC, MDP, FG and AM designed the study; GP, MC, MDP, FG, PB and AM reviewed the literature; MC, MDP and AM collected the data; FG, PB and AM performed the follow-up activity; GP, PN and SV performed the statistical analysis; GP, MC, PN, SV, MDP, PB, AM and SG wrote the manuscript. All the authors revised and approved the manuscript.
